# Bone Morphogenic Protein and Mesenchymal Stem Cells to Regenerate Bone in Calvarial Defects: A Systematic Review

**DOI:** 10.3390/jcm12124064

**Published:** 2023-06-15

**Authors:** Ricardo A. Torres-Guzman, Francisco R. Avila, Karla C. Maita, John P. Garcia, Gioacchino D. De Sario, Sahar Borna, Abdullah S. Eldaly, Alfredo Quinones-Hinojosa, Abba C. Zubair, Olivia A. Ho, Antonio J. Forte

**Affiliations:** 1 Division of Plastic Surgery, Mayo Clinic, 4500 San Pablo Rd., Jacksonville, FL 32224, USA; rtorresguzman@gmail.com (R.A.T.-G.); franciscorav93@gmail.com (F.R.A.); kmplasticsurgeon@gmail.com (K.C.M.); paulgarciamd@outlook.com (J.P.G.); giodesario@gmail.com (G.D.D.S.); saharborna1991@gmail.com (S.B.); abdullaheldali@gmail.com (A.S.E.); olivia.a.ho@gmail.com (O.A.H.); 2 Department of Neurosurgery, Mayo Clinic, Jacksonville, FL 32224, USA; quinones-hinojosa.alfredo@mayo.edu; 3 Department of Laboratory Medicine and Pathology, Transfusion Medicines and Stem Cell Therapy, Mayo Clinic, Jacksonville, FL 32224, USA; zubair.abba@mayo.edu

**Keywords:** models, animal, stem cells, tissue engineering, biocompatible materials, reconstructive surgical procedures, craniotomy, neurosurgical procedures, surgery, plastic

## Abstract

Background: The use of bone morphogenic protein and mesenchymal stem cells has shown promise in promoting bone regeneration in calvarial defects. However, a systematic review of the available literature is needed to evaluate the efficacy of this approach. Methods: We comprehensively searched electronic databases using MeSH terms related to skull defects, bone marrow mesenchymal stem cells, and bone morphogenic proteins. Eligible studies included animal studies that used BMP therapy and mesenchymal stem cells to promote bone regeneration in calvarial defects. Reviews, conference articles, book chapters, and non-English language studies were excluded. Two independent investigators conducted the search and data extraction. Results: Twenty-three studies published between 2010 and 2022 met our inclusion criteria after a full-text review of the forty-five records found in the search. Eight of the 23 studies used mice as models, while 15 used rats. The most common mesenchymal stem cell was bone marrow-derived, followed by adipose-derived. BMP-2 was the most popular. Stem cells were embedded in Scaffold (13), Transduction (7), and Transfection (3), and they were delivered BMP to cells. Each treatment used 2 × 10^4^–1 × 10^7^ mesenchymal stem cells, averaging 2.26 × 10^6^. Most BMP-transduced MSC studies used lentivirus. Conclusions: This systematic review examined BMP and MSC synergy in biomaterial scaffolds or alone. BMP therapy and mesenchymal stem cells in calvarial defects, alone, or with a scaffold regenerated bone. This method treats skull defects in clinical trials. The best scaffold material, therapeutic dosage, administration method, and long-term side effects need further study.

## 1. Introduction

Cranioplasty is a surgical procedure that aims to restore or repair skull discontinuity defects, following an injury or other surgical procedure, such as craniectomy or craniotomy. The rate of complications associated with cranioplasties in the United States is high. The overall complication risk is increased, especially in older age patients, as well as those undergoing delayed and large cranioplasties [1]. New alternatives have arisen, and, among them is the use of mesenchymal stem cells (MSCs). Adipose-derived stem cells (ADSCs) and bone marrow stem cells (BMSCs) have exhibit osteogenic ability both in vivo and in vitro. However, BMSCs have shown superior osteogenesis when compared to ADSCs [2].

The family of bone morphogenic proteins (BMP) are essential for osteogenesis. Clinical and preclinical studies have demonstrated the osteoinductive capacity of BMP-2 therapy in several intervention scenarios, such as bone defects, non-union fractures, spinal fusion, root canal surgery, and osteoporosis [3]. The United States Food and Drug Administration (FDA) approved the use of recombinant human BMP-2 (rhBMP-2) in several orthopedic and oral and maxillofacial applications, for instance spinal fusion surgery, tibial shaft repair, and maxillary sinus reconstructive surgery [4]. However, several side-effects of rhBMP-2 have been described. For instance, increased local inflammation response, leading to cervical spinal swelling and death, radiculitis, nerve injury, increased bone resorption rate, osteolysis, and ectopic bone formation due to leaking of rhBMP-2 from the implant site have been reported among others [4]. This review of the literature aims to assess the current state of the regenerative capacity of BMPs therapy in conjunction with mesenchymal stem cells (MSCs) in animal models to treat calvarial bone defects.

## 2. Methods

### 2.1. Eligibility Criteria

Our study included animal studies involving the use of BMP therapy and mesenchymal stem cells, either alone or seeded in a scaffold, to promote bone regeneration in a calvarial bone defect. Reviews, conference articles, book chapters, and in vitro studies in languages other than English were excluded.

### 2.2. Information Sources and Search Strategy

On 15 May 2023, two independent investigators conducted a computerized search using the following electronic databases: PubMed (1994-present); MEDLINE (1996-present); Embase (1988-present); Web of Science (1900-present); and CINAHL (1994-present). The MeSH terms “Skull Defect”, “Bone Marrow Mesenchymal Stem Cells”, and “Morphogenetic Proteins, Bone” were used.

### 2.3. Study Selection and Data Collection Process

**Figure 1:**
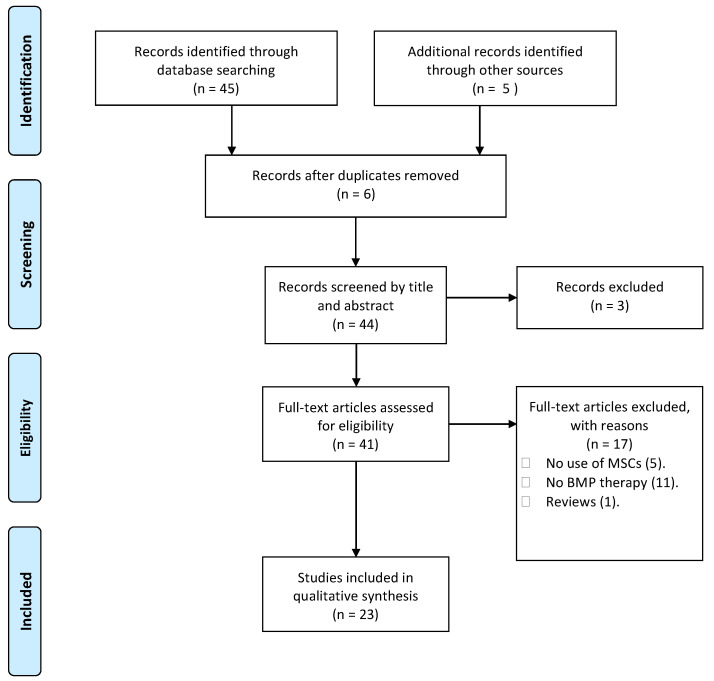
Study selection flow chart. Flow chart describing the study selection process, according to PRISMA guidelines [5].

Two researchers conducted the search and independently filtered studies based on titles and abstracts using the inclusion and exclusion criteria described above, and then they screened articles by reviewing the full text of the studies that had previously been filtered during the first stage. If no agreement could be reached, a third senior author designated whether the article should be included or excluded. Table 1 summarizes data from the selected articles. We used the Preferred Reporting Items for Systematic Reviews and Meta-Analyses (PRISMA) 2020 statement as the basis of our organization [5] (see Figure 1). No protocol was created because this systematic review was not registered.

### 2.4. Risk Bias Assessment

The bias risks of selected studies were assessed with the help of the ROBINS-I tool of the Cochrane Library for nonrandomized studies.

## 3. Results

### 3.1. Study Selection and Characteristics

Following a full-text review of the 45 records discovered in the search, we identified 23 studies that met our inclusion criteria and were published between 2010 and 2022. In eight of the 23 studies, mice were used as the animal model, while rats were used in 15. The most common type of mesenchymal stem cell was bone marrow-derived stem cells, followed by adipose-derived stem cells. The most commonly used BMP was BMP-2. BMP was delivered into cells via Embedded in Scaffold (13 total), Transduction (7 total), and Transfection (3 total). In each treatment, the number of mesenchymal stem cells used ranged from 2 × 10^4^ to 1 × 10^7^, with a mean of 2.26 × 10^6^. Lentivirus was the most commonly used vector in studies that transduced MSCs with BMP.

### 3.2. Area of Bone Regeneration and Bone Quality among the Included Studies

#### 3.2.1. Mice Models

**Figure 2:**
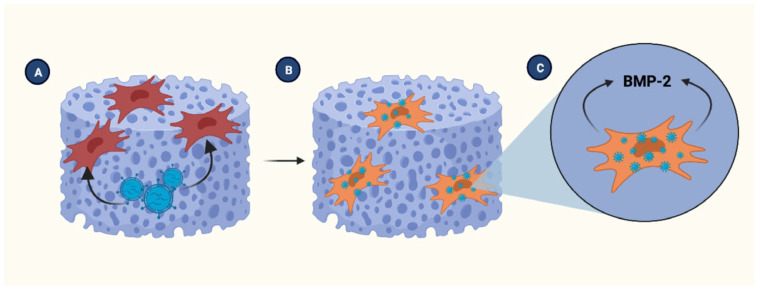
Transduced stem cells seeded in the scaffold. (**A**) BMSCs are seeded in the PLGA scaffold to then cultured with the viral vector to infect the cells. (**B**) Once transduced, BMSCs are capable to overexpress the required BMP. (**C**) BMP is transported from the intracellular space to the scaffold. BMSCs. Bone marrow-derived stem cells, BMP; Bone morphogenic protein, PLGA; poly(lactic-co-glycolic acid). Created with BioRender.com, accessed on 8 June 2023.

In 2011, Zhang et al. [27] used a silk fibroin scaffold to integrate adenoviruses encoding BMP-7 with human bone marrow-derived mesenchymal stem cells (hBMSCs). Then, they transplanted the scaffold into an animal model with a critical-sized skull defect. Compared to the control group, the silk scaffolds carrying adenovirus with and without hBMSCs significantly increased new bone formation (*p* < 0.05). Figure 2 depicts this example.

In 2014, Gao, X. et al. [9] compared the potential for bone regeneration between BMP-2 transduced hBMSCs and human muscle-derived stem cells (hMdSCs). The results demonstrate that the hBMSCs cohorts successfully regenerated bone within the defect, with no discernible differences in the newly formed bone. Furthermore, six weeks after the treatment administration, it was observed that both types of cells had produced mature bone tissue. However, it was observed that the hMdSCs underwent remodeling faster than the hBMSCs.

Vila, O.F. et al.’s 2014 study [26] investigated the effect of BMP-2 and fibrin-binding Platelet-Derived Growth Factor-BB (PDGF-BB) on cellular proliferation and osseous regeneration. Compared to the control treatment, BMP-2 and PDGF-BB demonstrated a greater propensity for enhancing bone and vascularization, albeit without a statistically significant increase in bone formation. In addition, incorporating human adipose-derived stem cells (hADSCs) into a fibrin scaffold improved vascular network connectivity, but it had no discernible effect on the volume of regenerated bone. Goldner’s Trichrome staining technique for osteoid has confirmed the findings mentioned above.

Aquino-Martinez, R. et al. [6] researched a groundbreaking technique for bone tissue engineering in 2016. Integration of gel/CaSO4 scaffolds, BMSCs, and minimal amounts of two osteoinductive agents (BMP-2 and Wnt3) was required. The study’s results indicate that using the composite scaffold in conjunction with the implantation of BMSCs resulted in more significant bone regeneration than the other conditions evaluated. In addition, it was discovered that scaffolds pretreated with a combination of Wnt3a and BMP-2 promoted enhanced recruitment of endogenous osteoprecursors and osteogenic potential. The previous observation suggests the potential application of a fusion of growth factor delivery techniques in bone tissue engineering, thereby accelerating the process of bone regeneration.

In 2016, Gohil, S. V. et al. [11] investigated the viability of utilizing calcium-hydroxyapatite (Chi-AHP) and Chi-AHP with rhBMP-2 for promoting bone regeneration. After four weeks, the Chi-AHP-implanted defect sites exhibited restricted cellular infiltration but no mineralized tissue formation or ALP/TRAP activity. At the defect sites where Chi-AHP with rhBMP-2 therapy was implanted, nascent osseous tissue emerged with mineralized regions. These osteoblasts tested positive for EYFP and a small amount of ALP/TRAP activity. At the eight-week mark, the Chi-AHP with rhBMP-2 therapy implanted side was observed to have mineralized new bone and residual injected gel, as well as non-mineralized marrow-like areas and vascular structures. Notably, however, the defect site had yet to undergo complete regeneration. This study’s results indicate that using Chi-AHP with rhBMP-2 therapy results in superior bone regeneration outcomes when compared to using Chi-AHP alone.

In 2019, Strecker, S. E., and colleagues [22] investigated the effectiveness rhBMP-2 and a nanocomposite scaffold material in promoting bone tissue regeneration. The immunostaining results indicate a significant increase in the cellular presence of Osterix and Osteocalcin. The participation of various cell types, including Mesenchymal stem cells, Osteoblasts, and Osteocytes, facilitates the repair process. At the four-week mark, osteoblasts and osteocytes were observable in the rhBMP-2-treated defects. In contrast, the nanocomposite-only treatments demonstrated a moderate osteocalcin expression increase. The empirical evidence suggests that using both rhBMP-2 and the nanocomposite scaffold effectively promotes osseous tissue regeneration.

In a recent study, Sun et al. [24] examined the osteogenic potential of two distinct modes of BMP-2 administration, emphasizing measuring the capacity for new bone formation. In this study, a comparison was conducted between the nTmGL group and the TmGL group. The first group consisted of a methacrylate gelatin scaffold loaded with hBMSCs and recombinant adeno-associated virus (rAAV) encoding BMP-2. In contrast, the latter group consisted of the same scaffold with ex-vivo transduced hBMSCs expressing BMP-2 using the same rAAV vector. The BMP-2 production of the nTmGL group is significantly higher than that of the other groups, as determined by in vitro studies (*p* < 0.01). As defined by bone volume (mm^3^) measurements using micro-CT-scan and three-dimensional reconstruction, both the nTmGL and TmGL groups demonstrated an increased capacity for new bone formation in vivo. However, the quantitative analysis revealed no statistically significant distinctions between the nTmGL and TmGL groups. The bone mineral density measurements of the TmGL group were significantly higher than the control groups (*p* < 0.01) and the TmGL group itself (*p* < 0.05).

The research conducted by Gao, X., and colleagues [10] in 2022 centered on creating an innovative bone tissue engineering technique to treat non-unions following bone fractures and segmental bone defects. The study utilized biocompatible materials as the delivery vehicle for bone growth factors. The results of the in vitro and in vivo experiments indicate that, among the 5 BMPs used, BMP-2 and BMP-7 had the most significant effects on promoting bone defect regrowth and bone regeneration. Through the administration of BMP-2 and BMP-7, osseous tissue was regenerated with a typical bone matrix and architecture. However, complete defect resolution was achieved after the six-week deadline. The experimental results demonstrate that the coacervate sustained release system can accommodate up to 2 g of BMPs for prolonged release.

#### 3.2.2. Rat Models

In 2010, Chuang, C.K. et al. [7] investigated the bone regenerative potential of hBMSCs transduced to express BMP-2 in immunocompetent rats with a calvarial defect of critical size. Four weeks after treatment application, histological analysis revealed that transduced hBMSCs formed numerous calcified bone matrices lined with osteoblast-like cells. However, by the 12th week, a significant portion of the calcified bone matrix had disappeared, and no additional bone formation was observed compared to the control groups. Similarly, at four weeks, CT scans of the transduced hBMSCs revealed the formation of bone islands in the central and peripheral regions of the defect, with a bone area (4.61.2%) that is double that of the control groups (scaffold only: 2.4 0.6% and mock-transduced hBMSCs: 2.6 0.7%). At 12 weeks, more bone was formed at the defect’s edge than in the control groups, but the difference was not statistically significant.

In the same year, Park, SH et al. [19] aimed to determine the efficacy of branched oligomerization of cell-permeable peptides (CPPs), such as Tat, which resulted in significantly enhanced adenoviral transduction of hBMSCs. The in vivo experiment revealed that neither the Matrigel scaffold alone, nor the Matrigel scaffold containing BMP-2 transduced hBMSCs with 0.4 mM Tat, significantly affected calvarial bone regeneration. In contrast, the Matrigel-containing hBMSCs group with 0.1 mM 4Tat resulted in more significant new bone formation that bridged the calvarial defect and substantially increased bone mineral content (*p* < 0.01). In addition, periosteum and fibrous connective tissue were primarily detected with hematoxylin and eosin and Masson’s Trichrome staining in calvarial defects of rats treated with Matrigel alone or Matrigel with transduced hBMSCs in the presence of 0.4 mM Tat. Rats treated with Matrigel containing BMP-2 transduced hBMSCs and 0.1 mM 4Tat developed cortical bone that was thicker and better organized.

To repair critical size defects in rat calvaria in 2010, Stephan, S.J. et al. [21] used an injectable biopolymer of chitosan and inorganic phosphates seeded with BMSCs and a BMP-2. The results demonstrated that this combination produced bone that could be detected histologically and with computed tomography, proving the viability of this method for minimally invasive delivery of a bone-forming construct. Compared to the control, gel alone, BMP-2/gel, and BMSC/gel, the percentage of bone regeneration by defect area as measured by micro-CT and the bone volume at eight weeks were significantly more significant in the BMP-2/BMSCs/Gel group (*p* < 0.05). In addition, the experiment revealed that defects containing chitosan gel, bone morphogenetic protein, and mesenchymal stem cells resulted in the most bone formation with the osteoid and gel cells. Immunohistochemical staining was used to confirm the presence of viable stem cells in the defect.

Terella, A. et al. [25] sought to evaluate the osteoconductive and osteoinductive properties of two poly (ethylene glycol) (PEG) scaffolds embedded with BMP-2 and mesenchymal stem cells in a significant, non-healing calvarial defect. The researchers hypothesized that, compared to the negative control, PEG scaffolds would be osteoconductive and that biofactors and mesenchymal stem cells would increase osteoinduction, thereby enhancing bone regrowth. poly (ethylene glycol) protease sensitive (PEG-MMP) and PEG-MMP + BMP2 significantly increased bone growth compared to controls. poly (ethylene glycol)-diacrylate (PEG-DA) inhibited bone formation, irrespective of biofactor or rat mesenchymal stem cells. There was no effect of rat mesenchymal stem cells on bone regeneration.

He, X. et al. [12] evaluated a chitosan/alginate/hydroxyapatite scaffold as a carrier for BMP-2 and its capacity to promote stem cell differentiation and bone formation in 2014. It demonstrated that the CAH/B2 scaffold had delayed BMP-2 release kinetics compared to collagen gel, enhanced stem cell differentiation, and exhibited no cytotoxicity. Analysis of in vivo bone formation revealed that the CAH/B2 scaffold induced more bone formation than other groups, indicating that it is a promising strategy for bone regeneration.

In 2014, Jin, H. et al. [14] demonstrated that implantation of BMP-2 gene-modified BMSC cell sheet (BMP/CS) or EGFP gene-modified BMSC cell sheet (EGFP/CS) to defects at both four and eight weeks resulted in significantly greater bone formation in the BMP/CS group than in the other groups. Within four weeks, micro-CT images revealed that the BMP/CS group demonstrated the most significant reduction in defect size. At eight weeks, image-pro Plus software (Image-Pro Plus 6.0; Media Cybernetics, Rockville, MD, USA) analysis revealed that the BMP/CS group had a significantly larger area of bone formation (57.9%6.5%) than the control group (35.6%3.3%) and the EGFP/CS group (48.9%2.1%). H&E histological staining at both four and eight weeks demonstrated that the BMP/CS group had significantly more new bone formation than the EGFP/CS group and the control group in the original defect margin.

Lee J.H. et al. [17] sought to determine the effects of combining rhBMP-2 with various growth factors on osteoinductivity in vitro and in vivo. This study investigated the effects of different combinations of growth factors, including epidermal growth factor (EGF), fibroblast growth factor (FGF), platelet-derived growth factor (PDGF), and vascular endothelial growth factor (VEGF), on calvarial defect models. Two weeks after surgery, the EGF combination group had the highest ratio of the new bone surface, while the FGF combination group had the highest percentage at six weeks. The ACS group had significantly lower levels of new bone surface ratio at two and six weeks and a higher bone volume percentage at six weeks. In addition, the ACS group had significantly greater specific surface area and lower anisotropy. The EGF combination group outperformed the other groups regarding calvarial defect improvement.

To repair critical-sized calvarial defects, Li, L. et al. [18] developed a novel dual-drug-loaded nanofiber scaffold with BMP-2 encapsulated in bovine serum albumin (BSA) nanoparticles (NPs) and dexamethasone (DEX) co-electrospun into the scaffold. This study investigated the effect of BMP-2 and DEX on calvaria repair in rats implanted with nanofiber scaffolds. The combination of BMP-2 and DEX resulted in a level of bone repair that exceeded 70%, which was significantly higher than the other groups tested. In addition to testing the apparent repair area and mean gray values, the results were confirmed. Compared to pure poly(ε-caprolactone)-co-poly(ethylene glycol) (PCE), NPs/PCE, the control, and single-drug-loaded nanofiber scaffolds, dual-drug-loaded nanofiber scaffolds BSA nanoparticles (BNPs) (BNPs/DEX/PCE) exhibit the highest osteogenesis capacity and bone repair ability for calvarial defect repair. According to histomorphometric, X-ray, H&E staining, and immunohistochemical staining analyses, the dual-drug-loaded scaffold (BNPs/DEX/PCE) was able to recruit MSCs, induce differentiation, degrade scaffolds, and form more mineralized bones than other groups.

Subbiah R. et al. [23] investigated a novel dual growth factor delivery system comprised of poly(lactic-co-glycolic acid) nanoparticles and alginate microcapsules containing Bone Morphogenetic Protein 2 and Vascular Endothelial Growth Factor. This study investigated, using three-dimensional collagen scaffolds, the promotion of dual growth factor release and osteogenic differentiation of umbilical cord blood-derived mesenchymal stem cells (UCB-MSC). To create an optimal microenvironment for the stem cells, both stem cells and mesenchymal condensates (MC) were seeded onto the scaffolds. After 28 days of in vitro culture, the cells’ osteogenic efficiency and calcium content were evaluated. The highest osteogenic efficiency and calcium content were observed in the MC group, indicating that three-dimensional collagen scaffolds can be used to facilitate UCB-MSC osteogenic differentiation.

Du, M. et al. [8] analyzed the biological behavior of bone marrow mesenchymal stem cells (BMMSCs) pretreated with bFGF or BMP-2 and the bone regeneration process induced by bFGF and BMP-2 loaded acellular dermal matrix (ADM) membrane. Compared to BMP-2, the proliferation and osteogenic differentiation capabilities of BMMSCs treated with bFGF were greater. At two weeks, two-fold more CD34/CD90 + MSCs were observed in the bFGF-ADM group than in the other treatment groups. At eight weeks, bFGF-ADM and BMP-2-ADM promoted comparable bone regeneration and more significant formation of new bone than ADM alone and blank control.

Hsieh, M. K. et al. [13] studied BMP-2 loaded onto a Matrigel scaffold was utilized to investigate the potential of non-viral gene therapy to promote bone healing. Macroscopic, X-ray, and micro-CT images were used after 12 weeks to evaluate the osteogenic potential of a rat calvarial defect model. Compared to the gel-only group (group A), adding BMSCs or TransIT/BMP-2 BMSCs caused an increase in bone. According to micro-CT analysis, a moderately mineralized callus partially bridged the defect region in group B and nearly completely bridged the defect region in group C. At 12 weeks postoperatively, histologic examinations revealed callus formation in groups B and C’s H&E-stained defects, with more osteoid deposition in group C than in group B. In Masson’s trichrome stain, group A had minimal osteoid and large amounts of un-resorbed gel, group B had thick fibrous connective tissue with thin bone-like tissue, and group C had bone-like tissue nearly filled with neovascularization. Group C demonstrated the highest levels of gel resorption and new bone formation.

Kuttappan S. et al. [16] investigated the ability of a nanocomposite fibrous scaffold loaded with fibroblast growth factor 2 (FGF-2), vascular endothelial growth factor (VEGF), and BMP-2 to promote vascularization and bone regeneration in a defect of critical size in the calvarium. This study determined that using growth factors to regenerate cranial defects is preferable to not using growth factors, mainly when both growth factors are used simultaneously. At four and twelve weeks, the dual growth factor loaded groups outperformed the other groups. In addition, all groups demonstrated an increase in the vascularization of the defect, with the growth factor-loaded groups showing the most vascularization. According to histology and histomorphometry, growth factor-loaded groups exhibited enhanced cellular infiltration and more outstanding organization of newly formed bone with collagen deposition.

Shao, N. et al. [20] investigated a new strategy for covalently bonding bioactive molecules onto inorganic hydroxyapatite (HAp) to improve compatibility between organic and inorganic components and endow bone composites with sustained bioactivity. Biocompatibility and bone regeneration therapy tests on the developed composite of gelatin methacrylamide (GelMA), four-armed PEG methacrylamide (four-armed PEGMA), and nano-hydroxyapatite containing bone morphogenetic protein-2 (nHAp-BMP-2) were successful. Furthermore, using the composite to treat a rat calvarial defect model produced the best results regarding the new bone volume and the highest ratio of new bone.

In 2019, Kong, Y. et al. [15], using micro-CT imaging with three-dimensional reconstruction, investigated the effects of microcapsules on in vivo bone defect repair. Bone mineral density, bone volume, and trabecular thickness were compared to the control and PLLA microspheres in alginate microcapsules (Alg) groups. The values improved significantly in the BMP/MSC/Alg, BMP/Alg, and MSC/Alg groups. Furthermore, compared to the control group, the BMP/MSC/Alg group had the highest BMD values, while the MSC/Alg group had the greatest bone mineral density improvement. These findings suggest that microcapsules may promote bone formation and repair bone defects.

Furthermore, this study found that microcapsules containing BMP and mesenchymal stem cells effectively repair calvarial defects. Various stages of repair efficacy were observed, with the MSC/Alg and BMP/MSC/Alg groups displaying the most significant quantity and maturity of newly formed bone, with the defect gap nearly wholly filled. In addition, it was discovered that the osteoblasts recruited in both groups had abundant collagen fibers and superior osteogenic capacity.

Zhou, C. et al. [28] investigated the effects of a composite tissue-engineered bone material consisting of bone mesenchymal stem cells, a bone morphogenetic protein gene lentiviral vector, and P3HB4HB thermogel on calvarial skull defects in rats. P3HB4HB is a fourth-generation synthetic polymer widely used in tissue engineering due to its high biocompatibility, superior mechanical properties, and suitable biodegradability. The results demonstrated that the material was compatible with cell tissue and stimulated the expression of osteogenic factors (RUNX2, OCN, OPN, and OSX). In addition, rats with calvarial defects exhibited a significantly increased capacity for tissue repair, significantly reducing pathological injury and increasing collagen fiber production. This suggests that tissue engineering could be used to regenerate bone defects using composite bone repair material.

## 4. Discussion

### 4.1. Clinical Implications of BMP-2 and Bone Regeneration

When considering bone regeneration, it is important to understand the different types of BMPs (bone morphogenetic proteins) and how they may impact the healing process. BMP-2 and BMP-7 are the most commonly studied BMPs, promising to promote bone growth and regeneration. However, BMP-2 is more effective in promoting osteogenesis (forming new bone tissue), while BMP-7 promotes chondrogenesis (forming cartilage). We found that 17 of our included studies were administered BMP-2, one was administered multiple BMPs therapy, one was administered BMP-7, and three were administered rhBMP-2. One study administered BMP-9. BMP-2 is one of the most potent BMPs for promoting the osteogenic differentiation of BMSCs in vitro and in vivo [29]. Although BMSCs are the predominant source of seed cells for bone engineering, their limited cell number and invasive harvesting process limit their application in clinical fields.

Preclinical data, efficacy, and feasibility of studies in animals served as backbones for the clinical trials that intend to use recombinant human BMP-2 (rhBMP-2) for several bone diseases, such as: bone defects, non-union fractures, spinal fusion, root canal surgery, and osteoporosis [3]. At the endosteal bone surface, rhBMP-2 affects bone resorption and formation, which results in the downregulation of Runx2, collagen type I, and Wnt signaling. At the periosteal surface, BMPs promote endochondral bone formation [30]. Nonetheless, several side effects have been reported by the FDA with the use of rhBMP [4].

BMP-2 has several clinical side effects [31], including ectopic bone formation, osteoclast activation, osteolysis, and subsidence. However, optimizing the use of BMP-2, such as by limiting the dosage or ensuring the absorbable collagen sponge is positioned correctly, can help mitigate some of these side effects. Bone cyst formation with BMP-2 can be attributed to the treatment’s pro-adipogenic effects, which reduce the overall bone quality. In addition, BMP-2 has been linked to various inflammatory and wound complications, such as epidural hematomas, wound dehiscences, postoperative fevers, and bleeding. Urogenital complications, such as retrograde ejaculation and bladder retention, have also been associated with using BMP-2. Lastly, even though clinical studies have not generally been able to detect a connection between BMP-2 and carcinogenesis, basic biological studies indicate that aberrant BMP activity plays a role in carcinogenesis.

BMP-2 causes inflammation, osteoclast activation, and adipogenesis in animal models [31]. Using supraphysiologic doses of BMP-2 can result in structurally abnormal bone formation, while elevated TNF levels inhibit osteoblastic differentiation and bone formation. Additionally, BMP-2’s adipogenesis induction can reduce the bone quality it forms.

### 4.2. Scaffolds in Tissue Engineering

The last decade of research on extracellular matrices and scaffold applications in osteogenesis has centered primarily on elucidating the composition of ECM and determining how ECM peptides can promote osteoblast differentiation. In addition, decellularized extracellular matrix scaffolds and three-dimensional nanofiber scaffolds have been developed and used to treat femoral defects in rats and promote cartilage and bone tissue regeneration [32].

Since 2002, research into hydrogels’ use to differentiate Mesenchymal Stem Cells (MSCs) into osteoblasts has increased substantially. Utilizing synthetic hydrogels, such as polyethylene glycol and poly (vinyl alcohol), an osteoid matrix has been successfully created. In addition, hydrogels with photothermal effects, biomimetic self-assembled peptide hydrogels, and electrically conductive hydrogels, utilizing graphene nanoparticles and polyaniline, have gained interest recently. These hydrogels have been observed to enhance osteoconductivity, mechanical strength, the expression of osteogenic genes, and the differentiation of MSCs into osteoblasts [32].

In the past two decades, researchers have investigated using various scaffolds and biomaterials to facilitate drug delivery and bone tissue engineering using scaffolds and biomaterials. Composite scaffolds, nanobiomaterials, and autologous platelet-rich fibrin have been identified as effective drug delivery systems that can accelerate tissue regeneration and healing by promoting osteogenesis. In addition, exosomal delivery has been studied for cartilage regeneration more recently. These osteogenic mechanisms associated with drug delivery can improve the efficacy of therapeutic applications [32].

The additive manufacturing technique of three-dimensional printing is beneficial for clinical applications. Fused Deposition Modeling, Stereolithography, and PolyJets are utilized to achieve the desired material properties. In addition, coating enhancements, such as the polydopamine/hydroxyapatite coating, have been added to this technology to improve scaffold stiffness, biocompatibility, and osteogenic differentiation potential [32].

We discovered that various scaffolds, including silk fibroin, fibrin, gel/CAOS4, calcium-hydroxyapatite, and methacrylic gelatin, were utilized in mouse model studies. In contrast, we discovered that the scaffolds discussed in the text were utilized in studies involving rat models. These scaffolds include Matrigel, chitosan/alginate/hydroxyapatite, poly(lactic-co-glycolic acid) nanoparticles, alginate microcapsules, three-dimensional collagen scaffolds, acellular dermal matrix, and poly-3-hydroxybutyrate 4-hydroxybutyrate thermogel. To promote regeneration and healing, each of these scaffolds is combined with BMP-2, FGF-2, PDGF, VEGF, BSA nanoparticles, mesenchymal condensates, four-armed PEGMA, gelatin methacrylamide, and other growth factors.

Each material has different advantages in the process, such as relatively higher osteogenic efficiency and bone growth, increased proliferation and osteogenic differentiation, more resorption, or increased vascularization capabilities. However, due to the numerous variables in each study, it is difficult to determine the advantages of each scaffold material by itself, such as relatively higher osteogenic efficiency and bone growth, increased proliferation and osteogenic differentiation, more resorption, or increased vascularization capabilities.

### 4.3. Role of MSCs in Bone Injury Healing

As described in the studies, mice and rats were the most common animal models used. This is because rodent (particularly rat) models are extraordinarily useful for conducting basic skeletal research and are dependable, cost-effective alternatives to dogs and nonhuman primates [33]. In addition, numerous studies have demonstrated that transplanted MSCs can promote in vivo wound healing by secreting cytokines, chemokines, and growth factors. In conjunction with the directed differentiation of MSCs into damaged tissue, this paracrine property is essential for tissue repair and regeneration [34]. MSCs extracted from the periosteum and bone marrow are the primary sources for bone formation and are always utilized in clinical skeletal repair [35].

**Figure 3:**
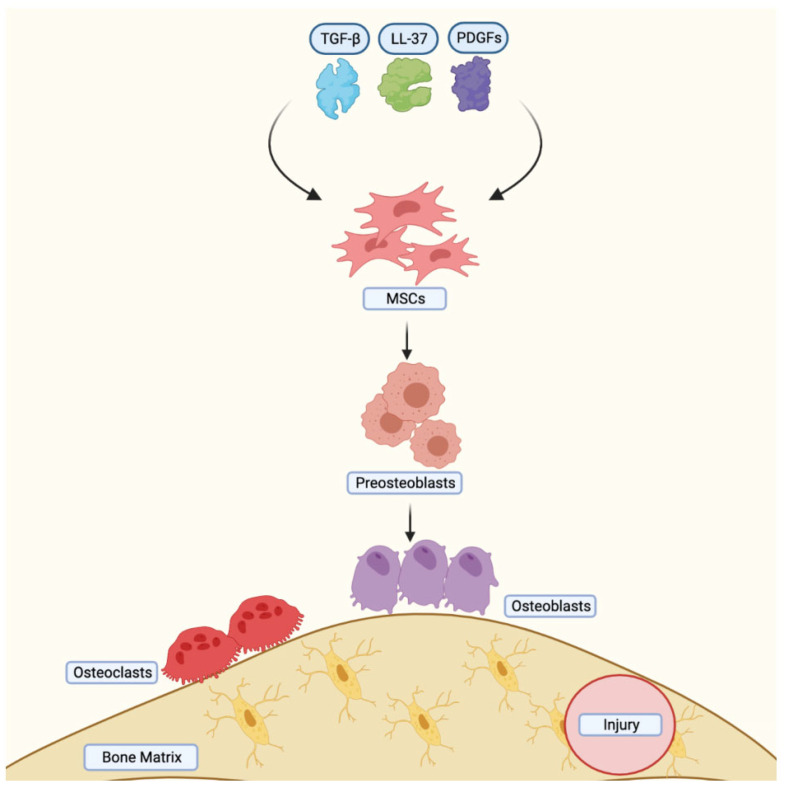
Mesenchymal stem cell migration during intramembranous ossification. The release of TGF-β, LL-37, and PDFG after bone resorption in addition to the expression of MMP promote migration of MSCs to the bone surface. MSCs differentiate into preosteoblasts. GTPase, BMP2, and RANKL accelerate migration to the bone surface. MSCs: Mesenchymal stem cells, TGF-β: Transforming growth factor-β, PDGFs: Platelet-derived growth factors, MMP: Matrix metalloproteinases, GTPase: Guanosine triphosphatase, BMP2: Bone morphogenic protein-2, LL-37: human antimicrobial peptide cathelicidin LL-37. Created with BioRender.com, accessed on 8 June 2023.

The bones of the calvarium and cranial vault are created through intramembranous ossification [36]. However, endochondral and intramembranous ossification are frequently combined during bone formation following a bone injury [35]. In order to gain better understanding of the mechanisms involved in bone injury repair, this process has been replicated using MSCs (see Figure 3). MSCs migrate during intramembranous ossification due to bone reabsorption-derived human antimicrobial peptide cathelicidin LL-37, platelet-derived growth factors (PDGFs), Transforming growth factor- (TGF-), and bone matrix metalloproteinases. Simultaneously, MSCs differentiate into preosteoblasts, which proliferate and secrete ALP near the bone surface. Chemokines, such as BMPs, receptor activators of the nuclear factor kappa B ligand RANKL, and the high expression of guanosine triphosphatase (GTPase) by MSCs expedite their migration to the bone surface, where they mature into osteoblasts and form osteocytes embedded in the extracellular matrix [35,37].

**Figure 4:**
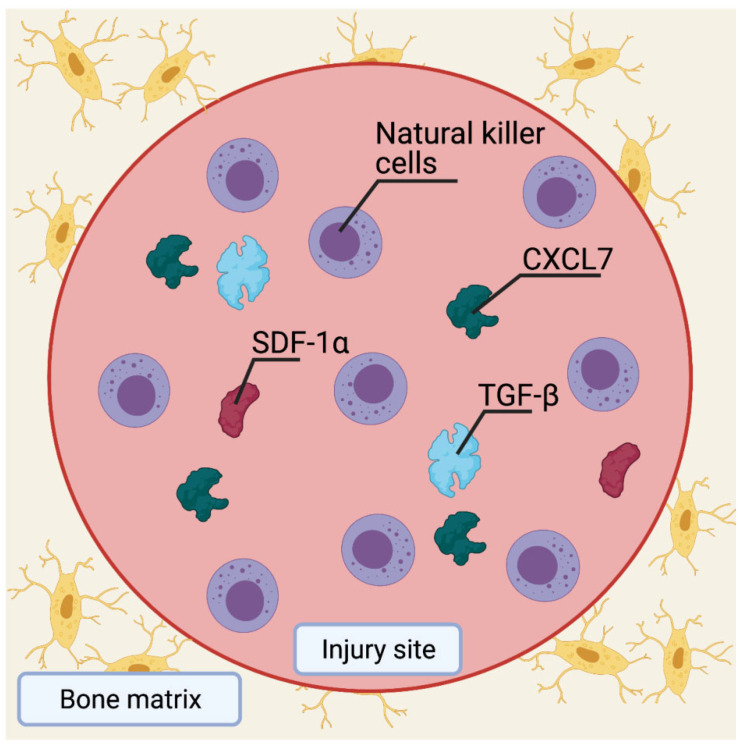
Migration of MSCs during bone fracture healing. Cytokines, such as TGF-β, LL-37, and PDGFs help migrate MSCs to the bone surface to differentiate into preosteoblasts. SDF-1α and CXCL7 are released from the injured bone site to boost preosteoblast migration. Preosteoblasts then differentiate into osteoblasts on the bone surface to assist in the healing process. MSCs: Mesenchymal stem cells, TGF-β: Transforming growth factor-β, PDGFs: Platelet-derived growth factors, LL-37: Human antimicrobial peptide cathelicidin LL-37, SDF-1: Stromal cell-derived factor 1. Created with BioRender.com, accessed on 8 June 2023.

In healing a bone fracture, the inflammatory response secretes LL-37, PDGFs, and TGF-, which aid MSC migration and differentiation into preosteoblasts [35]. Chemokines, such as stromal cell-derived factor 1 (SDF-1) and CXCL7 that are also released from the bone injury site, facilitate the migration of preosteoblasts to the bone surface, where they differentiate into osteoblasts (Figure 4). Understanding the pathways involved in bone healing has made it possible to use MSCs in synergistic therapy combinations for bone repair. BMPs, for example, promote the differentiation of MSCs into osteoblast and chondroblast lineages [30]. Even though BMP-8 has a higher osteogenic potential than the other BMPs, BMP-2, BMP-6, and BMP-9 may be the most potent inducers of MSC differentiation into osteoblasts. In contrast, the other BMPs primarily stimulate osteoblast maturation [38]. In vivo mouse fracture repair studies have shown that BMP-2 initiates the repair cascade with a peak in mRNA expression 24 hours after the injury [39]. In addition, BMP-2 regulates the expression of several other BMPs that are essential for the successful differentiation of MSCs into osteoblasts [40]. However, there is no significant difference between BMP-2, -4, and -7 in terms of their bone regenerative potential [41].

### 4.4. Limitations

There is a paucity of data in the literature regarding the in vivo studies on the mechanism of MSCs to repair/regenerate bone, specifically calvarial bone. Only studies published in English were included in our review. Additionally, no standardized dosage of MSCs is established for calvarial bone defect repair. The difference among characteristics of the animal model, as well as MSC donors, limit the comparison capacity among groups. Finally, the potential bias of misinterpreting data and results creates new variables to consider.

## 5. Conclusions

The studies described in this systematic review were conducted to determine the synergistic potential of BMP and MSCs when used alone or seeded in biomaterial scaffolds. The descriptive findings indicate that BMP therapy and mesenchymal stem cells in calvarial defects, either alone or in conjunction with a scaffold, promoted bone regeneration. In clinical trials, the results show that this method can effectively treat skull defects. More research is needed to determine the optimal scaffold material, therapeutic dosage and administration methods, and long-term side effects.

## Figures and Tables

**Table 1:** Table of the characteristics in the included studies.

Authors and Year	Animal Model and Age	MSCs Type	Transduction/Transfection Method	BMP Therapy Delivery Method	Diameter Size of the Defect	Approximate Number of Cells Seeded	BMP Therapy	BMP Dose	Type of Scaffold Used to Seed MSCs	Endpoints
Aquino-Martinez, R. et al., 2016 [6]	Six to eight-week-old GFP transgenic BALB/c mice	BMSCs	Embedded in scaffold	Embedded in scaffold	5 mm	3.5 × 10^5^	BMP-2	2 nM	Gelatin/CaSO4 scaffold	5 Weeks
Chuang, C.K. et al., 2010 [7]	Female immunocompetent Sprague-Dawley rats. 10 weeks	BMSCs	Baculovirus	Transduction	8 mm	5 × 10^6^	BMP-2	Not reported	PLGA	4 and 12 weeks
Du, M. et al., 2017 [8]	Seven to eight-week-old, adult female Wistar rats	BMSCs	Embedded in scaffold	Embedded in scaffold	8 mm	4 × 10^4^	BMP-2	800 ng/mL	Acellular dermal matrix membrane	1 and 2 weeks
Gao, X. et al., 2014 [9].	Eight-week-old Male CD-1 nude mice	BMSCs	Lentivirus	Transduction	5 mm	1.5 × 10^6^	BMP-2	Not reported	Fibrin sealant	1, 14, 28 and 42 days
Gao, X. et al., 2022 [10]	Seven-weeks-old ICRSCID mice at	hMDSCs	Embedded in scaffold	Embedded in scaffold	5 mm	2 × 10^4^	BMP-2, 4, 6, 7, 9	50 ng/mL	Fibrin sealant	1, 14, 28 and 42 days
Gohil, S. V. et al., 2016 [11]	Col3.6Cyan (ECFP) mice	BMSCs	Embedded in scaffold	Embedded in scaffold	3.5 mm	3 × 10^6^ cells/cm^2^	rhBMP-2	2 ug	Chitosan thermogel	4 and 8 weeks
He, X. et al., 2014 [12]	Male SD rats at 8 weeks of age	BMSCs	Embedded in scaffold	Embedded in scaffold	8 mm	1 × 10^6^	BMP-2	200 ng/mL	Chitosan/alginate/hydroxyapatite	12 weeks
Hsieh, M. K. et al., 2018 [13]	Eight-week-old Sprague-Dawley male rats	BMSCs	*E. coli*/TransIT-2020	Transfection	8 mm	1 × 10^6^/mL	BMP-2	Not reported	Corning Matrigel basement membrane Matrix High Concentration	12 weeks
Jin, H. et al., 2014 [14]	Male Wistar rats	BMSCs	Cells in transfection media	Transfection	5 mm	2 × 10^5^	BMP-2	Not reported	polyethylenimine–alginate (PEI–al) nanocomposite	4 and 8 weeks
Kong, Y. et al., 2019 [15]	Eight-week-old Sprague-Dawley rats	BMSCs	Embedded in scaffold	Embedded in scaffold	5 mm	5 × 10^5^	BMP-2	0.5 ± 0.02 μg/mL	Sodium alginate microcapsules and polylactic acid (PLLA) microspheres	4 and 8 weeks
Kuttappan, S. et al., 2018 [16]	Four to five-month-old male Wistar rats	ADSCs	Embedded in scaffold	Embedded in scaffold	8 mm	5 × 10^4^	BMP-2	Not stated	Nanocomposite fibrous	4 and 12 weeks
Lee, J.H. et al., 2015 [17]	Male Sprague-Dawley rats	hADSCs	*E. coli*	Transfection	8 mm	2 × 10^3^–2 × 10^4^	rhBMP-2	Not reported	Collagen sponge	2 and 6 weeks
Li, L. et al., 2015 [18]	Female Sprague-Dawley rats	BMSCs	Embedded in scaffold	Embedded in scaffold	8 mm	2 × 10^4^	BMP-2	80 mg	Dexamethasone embedded PCE polymer	4 and 12 weeks
Park, S.H. et al., 2010 [19]	Male immunocompetent Sprague-Dawley rats. 6 weeks	BMSCs	rAd	Transduction	8 mm	5 × 10^5^	BMP-2	Not reported	Matrigel matrix	4 weeks
Shao, N. et al., 2018 [20]	Six-week-old Sprague-Dawley (SD) male rats	BMSCs	Embedded in scaffold	Embedded in scaffold	5 mm	5 × 10^4^	BMP-2	340–400 μg	Inorganic hydroxyapatite gel	12 weeks
Stephan, S.J. et al., 2010 [21]	Six to eight-month-old Sprague-Dawley rats	BMSCs	Embedded in scaffold	Embedded in scaffold	8 mm	0.3 × 10^6^	BMP-2	2 μg	Chitosan gel	4 and 8 weeks
Strecker, S. E. et al., 2019 [22]	Osterix-mCherry mice	BMSCs	Embedded in scaffold	Embedded in scaffold	4 mm	1.2 × 10^6^ cells/cm^2^	rhBMP-2	0.2 μg	Dextran-Dendrimer Hydrogel Nanocomposite	4 and 8 weeks
Subbiah, R. et al., 2015 [23]	Seven-week-old SD rats	UCMSCs	Embedded in scaffold	Embedded in scaffold	9 mm	2.5 × 10^6^	BMP-2	392 ± 18 ng	PLGA NP and alginate microcapsules	4 and 8 weeks
Sun, K. et al., 2020 [24]	SCID mice. Age not specified	BMSCs	rAAV	Transduction	5 mm	1 × 10^7^	BMP-2	Not reported	mGL hydrogel scaffold	6 weeks
Terella, A. et al., 2010 [25]	Albino male Sprague Dawley rats aged 10–11 weeks	Not specified	Embedded in scaffold	Embedded in scaffold	8 mm	1 × 10^7^	BMP-2	5–15 ng/80 uL	PEG-DA, and PEG-MMP	1, 4 and 8 weeks
Vila, O.F. et al., 2014 [26]	10-week-old SCID mice	hADSCs	Lentivirus	Transduction	3 mm	0.2 × 10^6^	BMP-2	Not reported	Fibrin matrix	6 weeks
Zhang, Y. et al., 2011 [27]	SCID mice. Age not specified	BMSCs	Adenovirus	Transduction	3 mm	1 × 10^6^	BMP-7	Not reported	Silk fibroin	4 weeks
Zhou, C. et al., 2020 [28]	Two-week-old Sprague-Dawley rats	BMSCs	Lentivirus	Transduction	5 mm	2 × 10^6^ cell/mL	BMP-9	Not reported	P3HB4HB thermogel	4 weeks

Abbreviations: BMP: Bone morphogenic protein; CT-scan: Computerized tomography scan; mGL: Methacrylated gelatin; H&E: Hematoxylin and eosin staining; hBMSCs: Human bone marrow-derived stem cells; hADSCs: Human adipose-derived stem cells; hMdSCs; Human muscle derived stem cells; nTmGL group: mGL scaffold loaded with BMP-2 and hBMSCs; PLGA: Poly-Lactic-Glycolic-Acid; rAAD: Recombinant adeno-associated virus; PEG-DA: Poly (ethylene glycol)-diacrylate; PEG-MMP: Protease sensitive PEG; rAd: Recombinant adenovirus; SCID: Severe combined immunodeficient; TmGL group: mGL scaffold seeded with hBMSCs transduced with BMP-2; hMDSCs: Human muscle derived stem cells; PCE: Poly(ε-caprolactone)-poly(ethylene glycol); UCB-MSCs: Umbilical cord blood-derived mesenchymal stem cells; PLGA NPs: Poly(lactic-co-glycolic acid) nanoparticles; nHAp-BMP-2: Nano-hydroxyapatite peptide covalently immobilized with BMP-2.

## Data Availability

Not applicable.
